# When Diagnosis Takes a Turn: A Case Series on Testicular Adrenal Rest Tumor/Leydig Cell Tumor

**DOI:** 10.7759/cureus.63014

**Published:** 2024-06-24

**Authors:** Maryum Arshad, Sahrish Khawaja, Maleeha Ayub, Ahmed Imran Siddiqi, Waqas Shafiq

**Affiliations:** 1 Diabetes and Endocrinology, Shaukat Khanum Memorial Cancer Hospital and Research Centre, Lahore, PAK; 2 Internal Medicine, Shaukat Khanum Memorial Cancer Hospital and Research Centre, Lahore, PAK

**Keywords:** tart (testicular adrenal rest tumor), testicular adrenal rest tumor, leydig cell tumor, congenital adrenal hyperplasia, precocious puberty (pp), testicular mass, cah (congenital adrenal hyperplasia), lct (leydig cell tumor)

## Abstract

Testicular adrenal rest tumor (TART) is a known complication of congenital adrenal hyperplasia (CAH) that simulates testicular germ cell tumors to the extent that they can pose a diagnostic challenge to treating physicians. In this case series, we have presented four patients with different clinical scenarios but all of them presented with a common symptom of bilateral testicular masses. Their clinical histories were strongly suggestive of CAH. Most of them were treated initially as cases of germ cell tumor (Leydig) as their clinical features were overlapping, posing a diagnostic challenge. The histopathological features of CAH and Leydig cell tumors overlap considerably. Diagnosis of CAH must always be kept in mind as a differential diagnosis in patients presenting with bilateral testicular swellings. Timely diagnosis of TARTs and CAH can help preserve testicular functions. Careful histopathological analysis can add to the clinical features of CAH and Leydig tumors to correctly diagnose these patients. Here, we discuss this diagnostic challenge in our four patients.

## Introduction

Congenital adrenal hyperplasia (CAH) is a rare autosomal recessive disorder that affects the adrenal glands. It has a prevalence of <0.1% (general population) and is caused due to defective enzymes in the cortisol production pathway [1]. It is caused by certain enzyme deficiencies, mostly 21 hydroxylase deficiency (in more than 90% of cases), affecting steroidogenesis and reduced cortisol production by adrenals that leads to overstimulation of adrenal glands by adrenocorticotropic hormone (ACTH) through feedback mechanism leading to its hyperplasia and results in excessive testosterone production.

Adequate cortisol is required for feedback inhibition of ACTH, and, unfortunately, testosterone, which is another end product of the pathway, does not have a feedback effect over ACTH, so its uninhibited stimulation is the real issue.

It can be of two types: (1) classical CAH is usually considered the severe form and is mostly diagnosed at birth and (2) nonclassical CAH is a milder form that is often diagnosed in adults [2].

Usually, women with non-classic CAH present with signs of excessive androgen production, including excessive body hair, early puberty, and irregular periods, sometimes with virilization, while in men, it can be precocious puberty, low height due to early puberty, large genitals, and sometimes testicular masses secondary to testicular adrenal rest tumors (TARTs) [3]. TART is a common complication in male patients with CAH that can lead to gonadal dysfunction and fertility problems, especially if it progresses to an advanced stage. TART has no malignant features and surgical removal is not warranted at early stages but the central location of tumors near mediastinum testes can lead to compression of seminiferous tubules resulting in obstructive azoospermia/testicular tissue distortion and irreversible damage [4], so in advanced stage, orchiectomy is unavoidable. It is often challenging to differentiate clinically TARTs and leading cell tumors since these are closely related and usually share the same morphological characteristics. TARTs require surgical intervention only in advanced cases when they cause severe pain, discomfort, or large hard masses, otherwise, medical management with steroids is recommended. In contrast, Leydig cell tumors (LCTs) are always surgically removed as 10% of them are malignant in nature [5].

## Case presentation

Case 1: Eight-year-old boy (presented in January 2020 and having ongoing follow-up)

The patient was diagnosed with CAH at the age of two years as he had early signs of virilization (increased size of genitalia, pubic hair, and deepening of voice). He was started on oral hydrocortisone but, unfortunately, it was stopped after six months without medical advice. At the age of nine years, he presented to a tertiary care hospital with bilateral enlarged hard testicular masses and underwent right-sided testicular orchiectomy. Histopathology reported a Leydig cell tumor. Later, he was offered contralateral orchiectomy as well and was referred to our setup (cancer hospital). He was seen by the endocrinology team. On clinical exam, he had coarse facial features, bilateral axillary and pubic hair, and a large stony hard left-sided testicular mass. He was advised biochemical workup for CAH. The results are presented in Table 1.

**Table 1:** Biochemical workup. FSH: follicle-stimulating hormone; ACTH: adrenocorticotrophic hormone; DHEA-SO4: dehydroepiandrosterone sulfate; AFP: alpha-fetoprotein.

Tests	Results	Reference range
FSH	<0.1 mIU/mL	0.7-11.1 mIU/mL
Total testosterone	555.31 ng/dL	300-1000 ng/dL
17-hydroxyprogesterone	>20 ng/mL	0.27-1.99 ng/mL
ACTH	1106 pg/mL	<=46.0 pg/ml
DHEA-SO4	106 ug/dL	80-560 ug/dL

Scrotal Ultrasound

**Figure 1:**
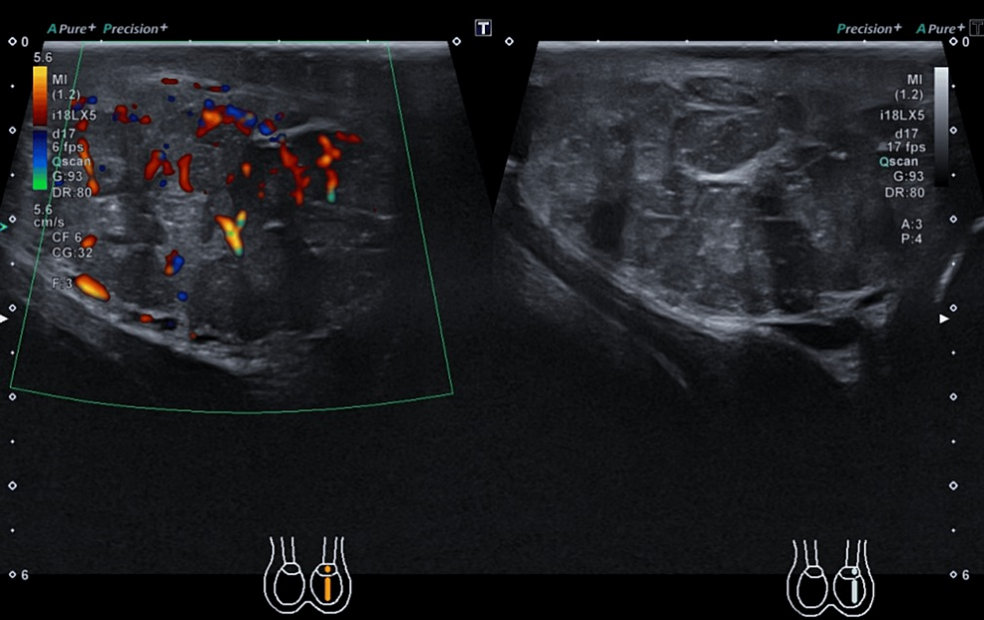
Ultrasound of the scrotum.

The left testis was entirely replaced by diffusely vascular, morphologically heterogeneous mass (Figure 1).

He was labeled as a likely case of TART with underlying CAH and started on prednisolone 2.5 mg once daily with a follow-up in six weeks with repeat blood tests and an ultrasound of the scrotum. Repeat blood tests at six weeks are presented in Table 2.

**Table 2:** Test and results of blood tests.

Tests	Results	Reference ranges/values
Testosterone	180.65 ng/dL	300-1000 ng/dL
17-hydroxyprogesterone	0.03 ng/mL	0.27-1.99 ng/Ml

Scrotal ultrasound showed stable testicular mass over the left side and changes in the right orchiectomy.

He is having regular follow-ups with the endocrine team with biochemical workups at three-month intervals and has adequate control. Unfortunately, he has a stony hard testicular huge mass, which has destructed testicular parenchyma. As the patient presented in advanced stages, so in the future, he will likely require left-sided orchidectomy as well to improve quality of life and symptomatic relief.

Case 2: 11-year-old boy (presented in June 2021 and ongoing follow-up)

An 11-year-old boy presented with bilateral painless testicular swellings for the last three months, gradually increasing in size. No associated fever was noted. Signs of precocious puberty from the last 1.5 to two years were noted by parents (shaving beards, axillary pubic hairs). On examination, bilateral huge testicular palpable masses were present. Hormonal profile is shown in Table 3.

**Table 3:** Hormonal profile. DHEA: dehydroepiandrosterone; ACTH: adrenocorticotropic; LH: luteinizing hormone; FSH: follicle-stimulating hormone.

Tests	Results	Reference values
17-hydroxyprogesterone	>20 ng/mL	0.27-1.99 ng/mL
DHEA	966 ug/dL	80-560 ug/dL
Total testosterone	1432 ng/dL	300-1000 ng/dL
ACTH	405 pg/mL	<=46.0 pg/mL
LH	<0.1 mIU/mL	0.7-11.1 mIU/mL
FSH	<0.1 mIU/mL	0.3-10.0 mIU/mL

*Scrotum* *Ultrasound *

**Figure 2:**
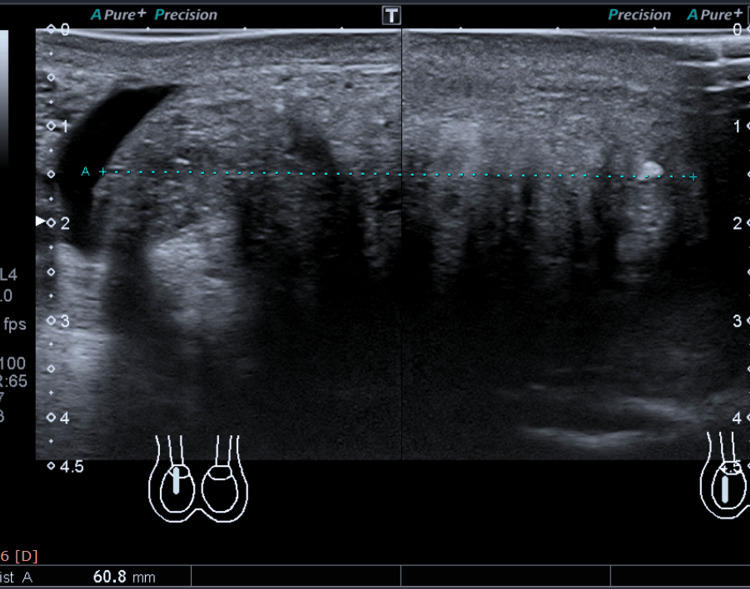
Pre-treatment scrotal ultrasound.

The right testis was completely replaced by a heterogeneous large mass measuring 6 cm in maximum dimension (Figure 2).

**Figure 3:**
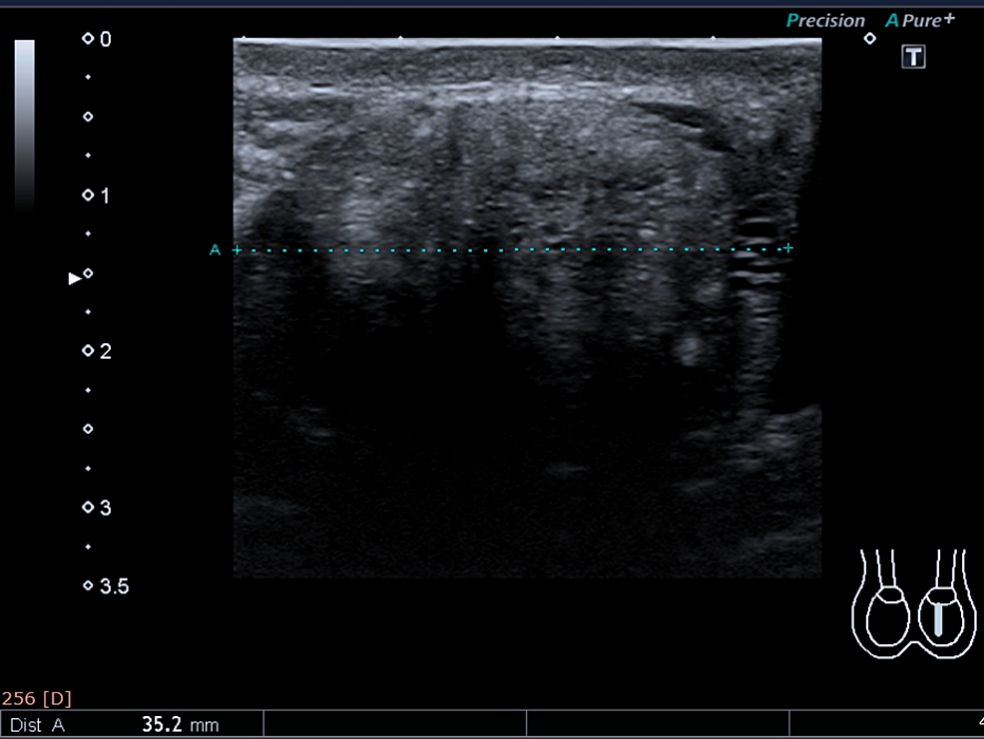
Pre-treatment scrotal ultrasound.

The mass showed internal vascularity on color Doppler. The left testis was also almost replaced by a heterogeneous large mass measuring 3.5 cm in maximum dimension (Figure 3).

Right testicular incisional biopsy tissue was submitted at Shaukat Khanum Memorial Cancer Hospital, and it was consistent with LCT, showing parenchymal infiltration by sheets of polygonal cells with abundant eosinophilic cytoplasm and round nuclei. Fine needle aspiration of the left testicular mass was consistent with atypical cells but could not be categorized further and a tru-cut biopsy was suggested. Synaptophysin was negative and CD56 was patchy positive. Reinke’s crystals were not seen. With strong suspicion of CAH causing TARTs, he was started on dexamethasone 0.5 mg once daily and was kept on follow-up. A persistent decrease in the size of testicular swellings was noted with steroid therapy, which was confirmed on ultrasound. A significant decrease in ACTH and 17-hydroxyprogesterone was also noted at the same time.

Ultrasound six months post-treatment showed the right testis heterogeneous mass with internal calcifications now measuring 3.3 compared to 6 cm previously. Left testis heterogeneous large mass measured 2.4 x 1.5 cm compared to 4.8 x 3.4 cm previously.

Currently, he is on regular follow-ups with the endocrine team with three-monthly biochemical profiles and usually six-monthly scrotal ultrasounds.

Case 3: 30-year-old male (presented in September 2021 and ongoing follow-up)

A 30-year-old male presented with a history of bilateral testicular swellings for the last six to seven years.

Scrotum Ultrasound

**Figure 4:**
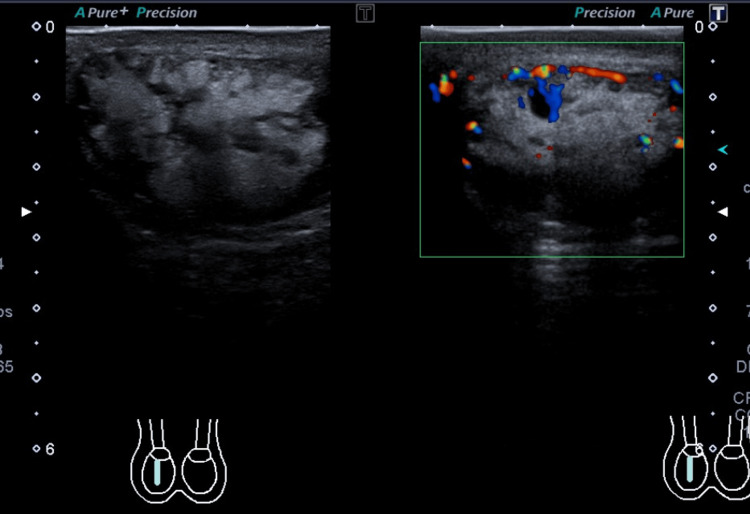
Scrotum ultrasound of the 30-year-old male.

Scrotal ultrasound showed the right testis measuring 36 x 32 x 21 mm, with a volume of 12.8 mL. The right testis demonstrated heterogeneous echogenicity (Figure 4).

The patient was seen by a urologist and underwent a right orchiectomy gross and a histopathological exam favored the diagnosis of LCT. Later, he underwent left orchiectomy and histopathology analysis showed a well-circumscribed tumor measuring 5 cm in size, with a brown, soft, gelatinous cut surface. It was composed of nests of plump tumor cells with bright, eosinophilic cytoplasm and mild atypia. CD56 staining was positive. Differentials were LCT/TARTs.

Post bilateral orchiectomy, he was referred to the endocrine team for hormonal replacement therapy. Biochemical workup was advised, which supported the diagnosis of CAH, as shown in Table 4.

**Table 4:** Biochemical workup. ACTH: adrenocorticotropic hormone; LDH: lactate dehydrogenase; AFP: alpha-fetoprotein.

Tests	Results	Reference ranges
17-hydroxyprogesterone	>20 ng/mL	0.27-1.99 ng/mL
ACTH	466 pg/mL	<=46.0 pg/mL
LDH	191 U/L	135-225 U/L
Beta human chorionic gonadotropin	<2 mIU/mL	<10 mIU/mL
AFP	1.04 IU/mL	<5 IU/mL

Tumor markers, including alpha-fetoprotein (AFP), beta human chorionic gonadotropin (HCG), and lactate dehydrogenase (LDH), were normal. Keeping in view the above scenario, he was labeled as a likely case of bilateral TARTs in the background of subclinical CAH. He was started on testosterone replacement as he had bilateral orchiectomies and steroid replacement with the aim of keeping ACTH levels within acceptable range to avoid pituitary hyperplasia.

Case 4: Nine-year-old boy (presented in November 2022 and ongoing follow-up)

A nine-year-old boy was diagnosed with CAH at two months of age (diagnosed with persistent vomiting and ambiguous genitalia, i.e., perineoscrotal hypospadias). He remained on hydrocortisone since infancy, but his dose was not adjusted, and biochemical control was not known for the last three to four years. He presented to the cancer hospital with a history of bilateral testicular masses with high suspicion of TARTs. He was seen by an endocrinologist who suggested biochemical control of CAH, which raised strong suspicion of bilateral TARTs. He was suggested to adjust steroid dosage and see treatment response in terms of reduction in the size of testicular masses after steroid therapy. His biochemical workup was in favor of CAH not biochemically controlled (Table 5).

**Table 5:** Test and results. ACTH: adrenocorticotropic hormone; DHEA-SO4: dehydroepiandrosterone sulfate.

Tests	Results	Reference ranges
Beta human chorionic gonadotropin	<2 mIU/mL	<5 mIU/mL
ACTH	1189 pg/mL	<=46.0 pg/mL
17-hydroxyprogesterone	>20 ng/mL	0.27-1.99 ng/mL
DHEA-SO4	911 ug/dL	80-560 ug/dL
Total testosterone	215.82 ng/dL	300-1000 ng/dL

Ultrasound of the scrotum showed bilateral testicular masses. His bone age studies showed 17 years of age and he had pubic and axillary hair requiring bimonthly shaving and somewhat mature facial features. His steroid dose was adjusted and was given six weekly follow-ups. Meanwhile, he was seen by the urology team and underwent a biopsy of the left testicular mass.

Histopathology saw no normal testicular tissue present in the biopsy, and without complete orchiectomy, it was not possible to comment on TARTs, so they suggested going ahead with left-sided orchiectomy for definitive tissue diagnosis.

A left-sided orchiectomy histopathology was done. Sectioning of the testis revealed a testicular parenchyma replaced by a golden-yellowish nodular lesion. The nodules have intervening fibrotic strands. Synaptophysin was positive.

A microscopic examination was conducted. Sections from the lesion in the testis revealed a tumor composed of diffuse sheets of medium to large polygonal cells with abundant eosinophilic cytoplasm distinct cell outlines, round nuclei, and prominent nucleoli considering feature differentials of TARTs/Leydig were suggested. Now he is labelled as having a case of bilateral TARTs with background classical CAH.

Currently, he is on regular follow-up with the endocrinology team and managed with oral steroids. His steroid replacement dosage is adjusted with monitoring of his biochemical profile, including 17-hydroxyprogesterone, ACTH, and testosterone levels.

## Discussion

In adult patients with CAH, TART is the most important cause of gonadal dysfunction and infertility [6]. The adrenal gland is near gonads during the embryological phase so few adrenocortical cells may rest within the rete testis but they involute in the early neonatal phase. In some percentage of people, adrenal rest cells remain in the testes and they get stimulated by ACTH and grow [7]. So, in patients with CAH, there is a problem in adrenal gland steroidogenesis, and hormone production is faulty, so there is stimulation of adrenal rest cells by ACTH by a feedback mechanism, so TART develops [8]. But if CAH is diagnosed and treated with steroids, blocking excessive ACTH production can avoid the development of testicular tumors and if developed, it can shrink their size. It is worth noting that TARTs often exhibit a positive response to corticosteroid therapy [9], rendering surgical intervention unnecessary. Clinically, patients with TART with background CAH may have a history of virilizing symptoms and bilateral involvement of testes. The occurrence of multimodality and bilaterality is frequently observed. Radiologically, ultrasound of the testes may help. These tumors may have a consistent hypoechoic appearance and possess delineated boundaries. Biochemically, there are raised 17-hydroxyprogesterone and ACTH levels. They are frequently observed in the hilar area of the testis. On gross examination, the observed nodules are characterized by a distinct yellow-to-brown coloration and are demarcated through strong fibrous bands. Therefore, it is imperative to differentiate between these two phenomena (Table 6).

**Table 6:** Differentiating features between TARTs and Leydig cell tumors. References [3,7,10-12]. CAH: congenital adrenal hyperplasia; TART: testicular adrenal rest tumor; AFP: alpha-fetoprotein; beta HCG: beta human chorionic gonadotropin.

Features	Leydig cell tumors	TARTs
History: (1) Site involvement. (2) Gynecomastia. (3) Precocious puberty. (4) Background history of CAH.	(1) Usually unilateral/rarely bilateral (3% cases). (2) Gynecomastia is usually present. (3) Precocious puberty is unrelated. (4) The background history of CAH is not present/if present, it can be a separate entity.	(1) Usually bilateral (more than 80%). (2) Gynecomastia is unrelated. (3) Precocious puberty. (4) The background history of CAH is there.
Biochemical markers: beta HCG/AFP level, 17-OH progesterone, 11-deoxycortisol, and testosterone will be raised	Levels: Elevated, normal	Levels: Normal, they will be raised
Histopathological analysis: Microscopic/histopathological/immunohistochemical findings	Size > 5 cm, infiltrative borders, cytological atypia, frequent mitoses (>3/10 high power fields), vascular invasion, and necrosis. Reinke crystals, which can be found in 25-40% of cases. Weak or negative reactivity for CD56, positive reactivity for the androgen receptor, and focal weak or negative reactivity for synaptophysin.	Lack of cytological atypia, low mitotic activity, dense fibrous septa, lymphoid aggregates, adipose metaplasia, and prominent lipochrome pigment. Reinke’s crystals will always be absent in TARTs. Diffuse and strong positivity for CD56, negative reactivity for the androgen receptor, and focal or diffuse strong reactivity for synaptophysin.

**Figure 5:**
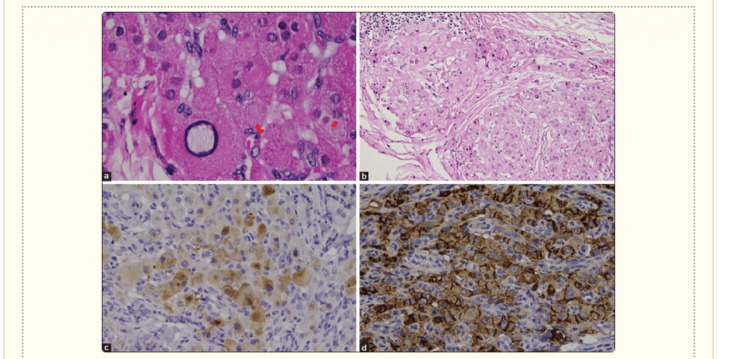
(a) Cells show mild nuclear pleomorphism with the absence of Reinke crystals and lack of mitotic activity. (b) Fibrosis surrounds the eosinophilic polygonal cells and lymphoid aggregates. (c) Patchy reactivity for synaptophysin. (d) Positive reactivity to CD56. Figure adapted from Ali et al. [12] under Creative Commons CC BY-NC-ND 4.0 license.

**Figure 6:**
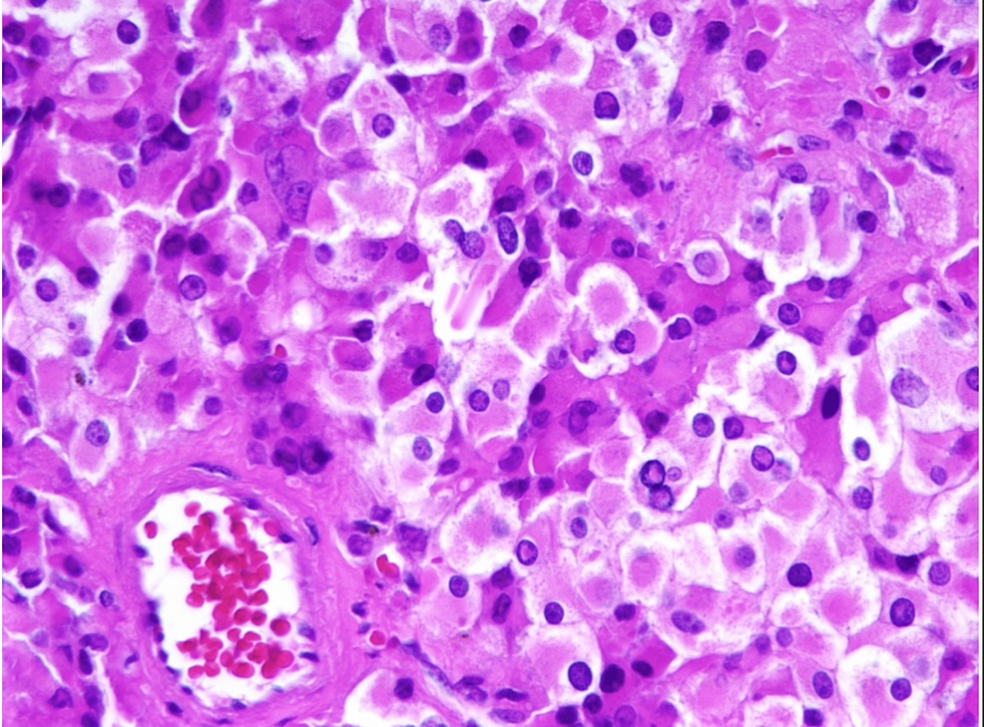
Histopathology image of testicular mass (Leydig cell tumor) showing polygonal cells with abundant eosinophilic granular cytoplasm, rounded nuclei, and intracellular Reinke crystals. Figure adapted from Chen and Aron. Leydig cell tumor [13].

TARTs exhibit a resemblance to adrenocortical tissue [10]. These tumors are characterized by the presence of large polygonal cells with abundant eosinophilic cytoplasm, which are grouped in strands or cords [11]. TART exhibits certain features that are more prevalent in comparison to LCT. The observed features are a lack of cytological atypia, minimal mitotic activity, the existence of dense fibrous septa, lymphoid clusters, adipose metaplasia, and notable lipochrome pigment. Reinke crystals, found in 25-40% of LCTs, are usually not observed in TARTs. The immunohistochemical research demonstrates that TART exhibits an extensive and strong expression of CD56, displaying either localized or widespread intense reactivity for synaptophysin (as shown in Figure 5). In contrast, LCT demonstrates focal weak to moderate or negative reactivity toward CD56 and focal weak or negative reactivity toward synaptophysin, while exhibiting positive reactivity toward the androgen receptor [7]. Reinke crystals are considered a potential indicator of Leydig cell malignancies (as shown in Figure 6).

LCT arises from the Leydig cells, which are found in the interstitial tissue of the testes. The diagnostic presence of Reinke crystals, observed in only 40% of patients, is a notable characteristic. Accurate identification of TART can be achieved with a comprehensive evaluation of clinical presentation, supported by the utilization of biochemical markers, imaging techniques, and histopathological analysis. In the field of medical imaging, ultrasound is commonly regarded as the preferred modality for diagnostic purposes and ongoing monitoring during therapeutic interventions. The importance of prompt diagnosis cannot be overstated, as the failure to address TARTs promptly may result in the deterioration of testicular function, leading to irreversible infertility. While TART is discernible and medically manageable, the tumors associated with TART are categorized into various phases. In the 1st stage, adrenal cells are within the testes with no clinical impact. Stages 2 and 3 can be treated medically but in stages 4 and 5, most of the testicular tissue is replaced by mass lesions distorting testicular structure and dense fibrosis. At this stage, medical therapy is not helpful and the patient usually requires surgical intervention [4].

The histological picture of LCT resembles very closely with TARTs and it is usually not possible to distinguish them histologically. Clinically, TARTs tend to present as bilateral, painful testicular masses as compared to unilateral, painless testicular masses in LCT. The treatment modality of TARTs (medical) and LCT (surgical) differs a lot.

## Conclusions

It is essential to differentiate between a benign condition that can be treated with pharmacological agents from a tumor that requires life-changing treatment including surgery.

CAH must be considered in differential diagnosis in all patients with testicular swelling. Overlapping histopathological features pose a challenge to conclusively differentiate between TART and LCT; however, clinical and biochemical features at a diagnostic stage can help to reliably differentiate between these two conditions.
